# Supplementary material for: Transcriptional overlap links DNA hypomethylation with DNA hypermethylation at adjacent promoters in cancer
Source: Sci Rep. 2021 Aug 30;11:17346. doi: 10.1038/s41598-021-96844-0 (PMC8405634; doi:10.1038/s41598-021-96844-0)
Supplement: Supplementary file 1 — Supplementary Information. [file 41598_2021_96844_MOESM1_ESM.pdf]

Supplementary Information for

Transcriptional overlap links DNA hypomethylation with DNA hypermethylation at adjacent promoters in cancer

Jean S. Fain, Axelle Loriot, Anna Diacofotaki, Aurélie Van Tongelen, and Charles De Smet

Corresponding author : Charles De Smet
Email:charles.desmet@uclouvain.be

This PDF file includes:

Supplementary methods
Figures S1 to S11
Tables S1 to S3
Original uncropped images
Supplementary References

Supplementary Methods

Processing of public RNA-seq raw data.
1) Read alignment: Fastq files from technical replicates, when available, were summed to increase sequencing depth. FastQC v0.11.8 was used for read quality control (1). Low-quality reads were discarded using Trimmomatics v0.38 (2). Resulting files were mapped to the genome GRCh38 using HISAT2 v2.1.0 with default parameters (3), and were converted to BAM files and indexed using Samtools v1.6 (4). Read alignments corresponding to splice junctions were visualized using the Integrative Genomics Viewer v2.3.68 (IGV) (5). *2) De novo transcriptome assembly and quantification of full-length referenced and unreferenced transcripts:* StringTie v1.3.4 assembling was applied to BAM files of the 26 LUAD cell lines, normal lung, and testis tissues (using -G option for GENCODE v27), and resulting GTF files were merged (--merge option) to produce a unified non-redundant set of transcripts (6). This merged GTF file was used for a re-quantification step in LUAD cell lines, lung, and testis. To this end, the BAM file for each sample was submitted to StringTie assembling (-e -B -G parameters), using the merged GTF as the reference transcriptome. Transcript expression levels are expressed as Transcripts Per Million (TPM).

Bioinformatics workflow for the identification of overlapped promoter hypermethylation.
1) Pooling of transcripts originating from the same promoter in LUAD cell lines: Transcripts originating from a TSS located less than 50 bp from each other were pooled for quantification, i.e. their expression level corresponded to the sum of individual transcript counts. For such pooled transcripts, the TSS most proximal to the 5' end was used as reference. *2) Selection of activated transcripts and potential overlapped promoters in LUAD cell lines:* Transcripts that are initially not expressed in normal lung (TPM < 0.1 in normal lung) were retained, and among these, those showing expression (> 10 TPM) in at least 1 to maximum 20 of the 26 LUAD cell lines were selected. Finally, transcripts overlapping another TSS, whatever its orientation, were identified. *3) Correlation between overlapping transcript expression and overlapped promoter methylation:* For each of the potential overlapped promoters, mean methylation levels in normal lung and LUAD cell lines (TSS +/- 400bp; promoters with less 3 CpGs within this range were discarded) was computed. Inclusion criteria for potentially hypermethylated overlapped promoters were: mean methylation level in normal lung < 40%; methylation data available in at least 10/26 LUAD cell lines; not all cell lines displaying > 40% methylation. Overlapped promoters that passed this sorting were then examined for potential correlation between their DNA methylation level and expression of the overlapping transcript in LUAD cell lines. Genomic loci displaying a correlation coefficient > 0.3 (*p*-value < 0.01), were selected. For overlapping transcripts corresponding to pooled transcripts (see step 1), the expression level was calculated on the basis of only transcripts variants that actually do overlap the downstream promoter. *4) Manual curation of overlapping transcript/overlapped promoter pairs.* To verify accuracy of TSS positions and transcript structures inferred from our transcriptome *de novo* assembly (see above), BAM files of LUAD cell lines were visualized using IGV (5). This led to a final selection of genomic loci, in which overlapping transcription was confirmed, and where position of the TSS of both the overlapping transcript and overlapped promoter could be validated.

Methylation-specific qPCR.
Bisulfite-treated DNA was submitted to a first PCR amplification with primer pairs recognizing DNA segments comprising either the *CT-GABRA3* or *GABRA3* promoter region, irrespective of their methylation status (primer pairs: CT-GABRA3 first PCR and GABRA3 first PCR, Table S2). Next,

nested qPCR were performed on 1:150 dilutions of the first amplification products with primer pairs recognizing the methylated (-MSM) or unmethylated (-MSU) sequence of the promoter region of either *CT-GABRA3* (primer pairs: CT-GABRA3-MSM, CT-GABRA3-MSU, Table S2) or *GABRA3* (primer pairs: GABRA3-MSM, GABRA3-MSU, Table S2). For qPCR, we used the qPCR Core kit for SYBR Green (Eurogentec, Belgium), with cycling conditions described in Table S2. Relative *CT-GABRA3* hypomethylation or *GABRA3* hypermethylation levels were determined by calculating the ratio of MSU / (MSU+MSM) or MSM / (MSU+MSM), respectively, which were inferred from the -∂CT values. Three technical replicates were performed. Genomic DNA of melanoma samples was obtained from Dr D. Rimoldi of the University of Lausanne.

Supplementary Figures

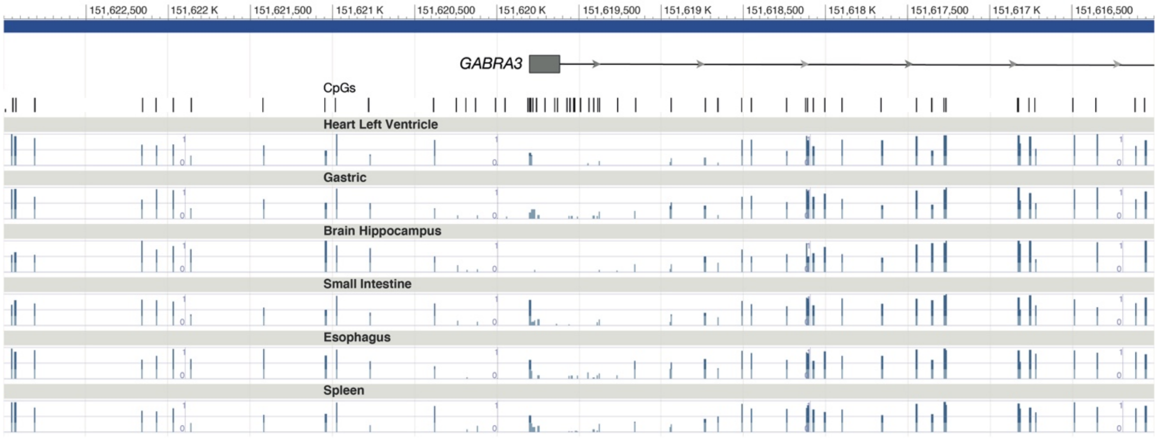

Fig. S1. CpGs within the 5'-region of *GABRA3* are mostly unmethylated in normal tissues. Bisulfite-seq data from different normal human tissues (all from males) were visualized with the NIH Roadmap epigenomics track viewer: histograms depict CpG methylation levels (fraction methylated). Position of CpG sites and of exon 1 of *GABRA3* are depicted above.

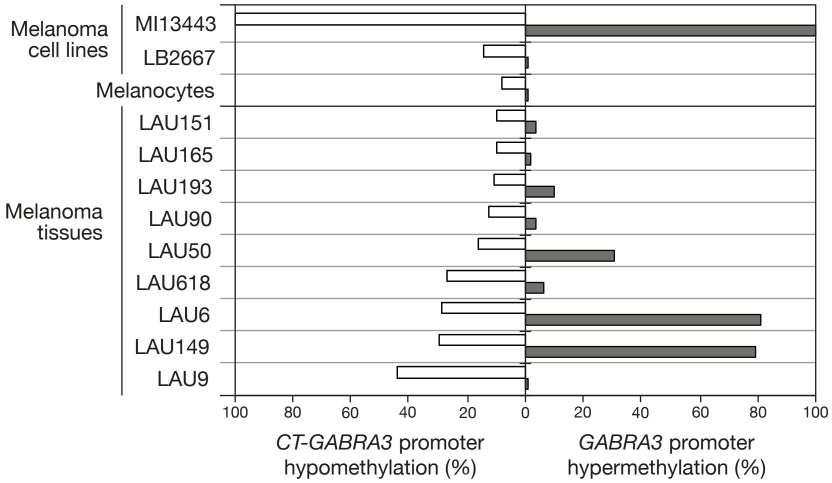

Fig. S2. Tendency for co-occurrence of *CT-GABRA3* promoter hypomethylation and *GABRA3* promoter hypermethylation in melanoma tissue samples. Methylation-specific qPCR (MS-qPCR) assessing *CT-GABRA3* and *GABRA3* promoter methylation levels was conducted on DNA obtained from 10 cutaneous melanoma tissue samples. Control samples include Mi13443 (*CT-GABRA3*+) and LB2667 (*CT-GABRA3*-) melanoma cell lines, as well as normal melanocytes (see detailed methylation status of *CT-GABRA3* and *GABRA3* promoters in these samples in Fig. 1B). Relative methylation levels represent the amplification ratio obtained from bisulfite-treated DNA with primers recognizing either the methylated (MSM) or unmethylated (MSU) promoter sequences. For *CT-GABRA3*, values are expressed as % hypomethylation (100 x MSU/MSU+MSM), whereas for *GABRA3* they are expressed as % hypermethylation (100 x MSM/MSU+MSM). Melanoma samples are ordered according to the *CT-GABRA3* hypomethylation level.

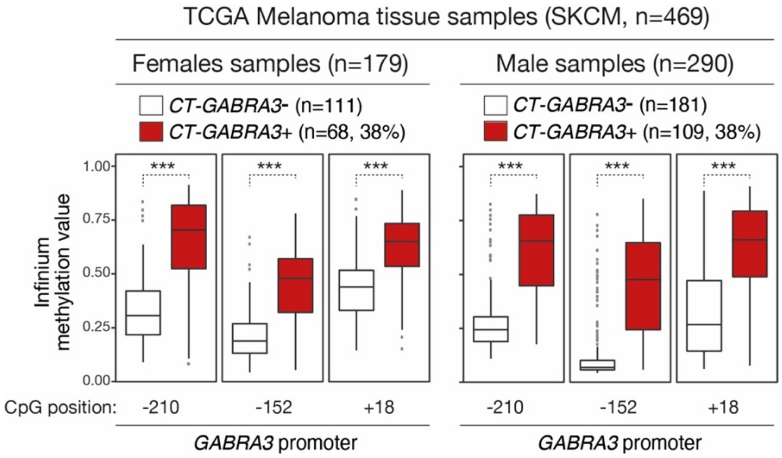

Fig. S3. Hypo/hypermethylation in the *GABRA3* locus is not affected by gender. Melanoma tissue samples from the TCGA were grouped according to gender and to the *CT-GABRA3* expression status (inferred from RNA-seq data). The frequency of activation of *CT-GABRA3* was identical in melanoma samples of male and female origin (38%). The methylation level of three CpG sites embedded within the *GABRA3* 5'-region (position relative to TSS) were determined in the different groups of melanoma samples through the analysis of Infinium methylation data (probe intensity ratio). Samples of both male and female showed significant association between *CT-GABRA3* activation and hypermethylation of CpG sites within the *GABRA3* promoter region. *** Welch's t-test, adjusted p-value <0.001.

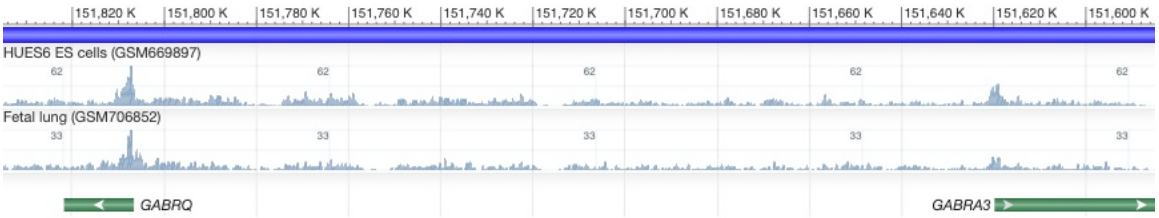

Fig. S4. *GABRA3* and *GABRQ* promoters are pre-marked with H3K27me3. A common characteristic of gene promoters that become hypermethylated in tumors is their initial association with the repressive H3K27me3 histone mark, which is often present since early embryonic stages. To determine if this is also the case for *GABRA3* and *GABRQ*, H3K27me3 ChIP-seq data from human ES cells and fetal lung were visualized with the NIH Roadmap epigenomics track viewer: histograms depict H3K27me3 enrichment. Chromosome (X) coordinates refer to the GRCh37.p13 genome assembly release.

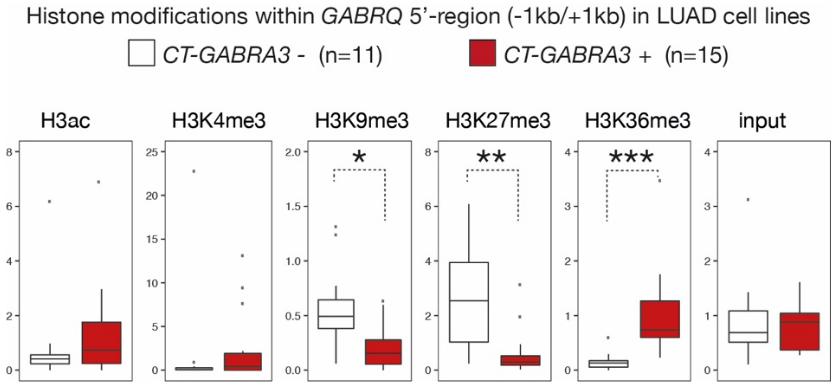

Fig. S5. *CT-GABRA3* transcription correlates with decreased H3K9me3/H3K27me3, and increased H3K36me3 within the 5'-region of *GABRQ*. (A) ChIP-seq results for each histone modification were analyzed for the *GABRQ* 5'-region (TSS +/- 1kb) in LUAD cell lines that do or do not express *CT-GABRA3*. Analysis was carried out as described in the material and methods section. *, **, and *** Mann Whitney test, adjusted *p*-value <0.05, <0.01, and <0.001, respectively.

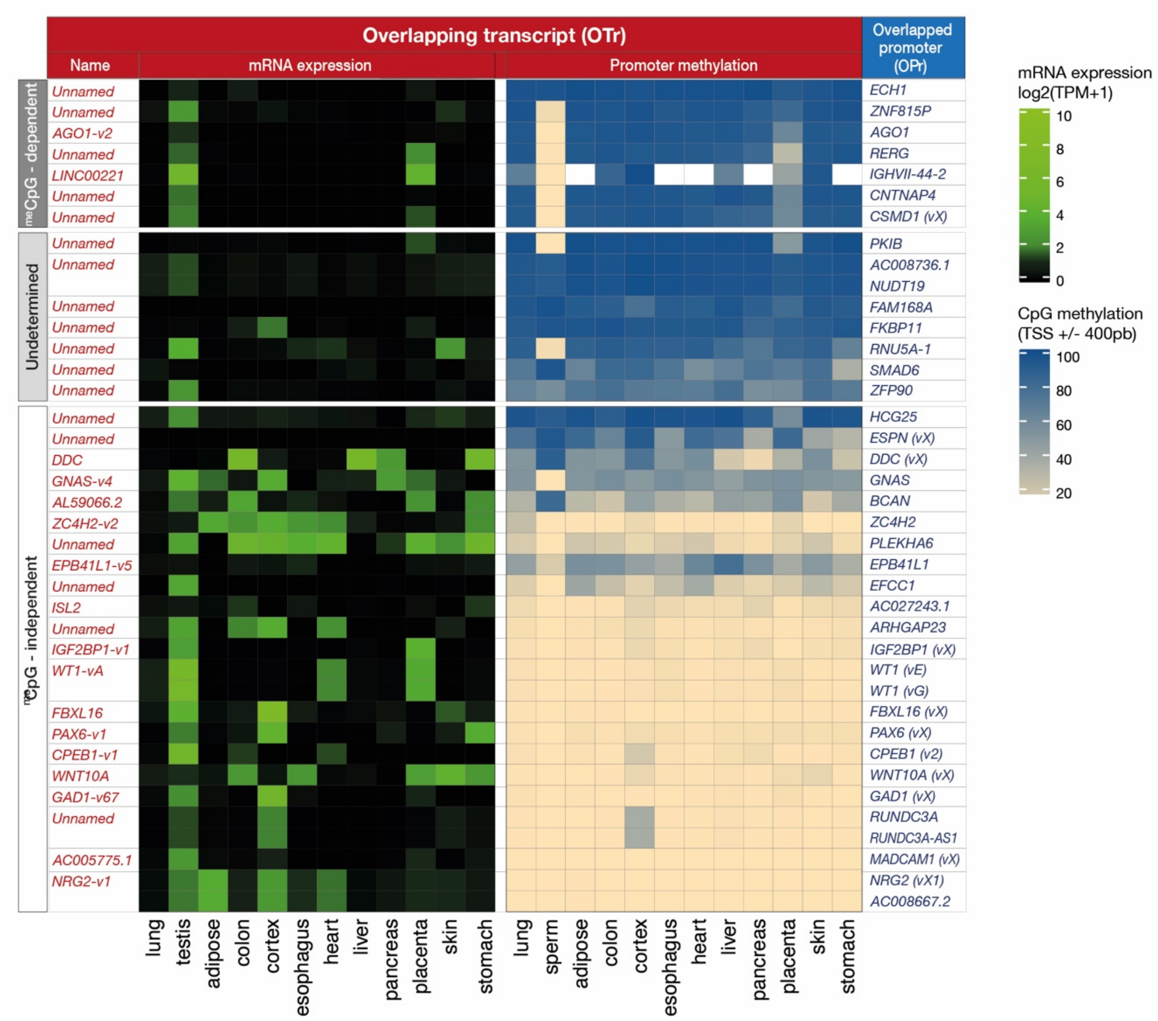

Fig. S6. Patterns of mRNA expression and mean promoter DNA methylation (TSS +/- 400bp) of overlapping transcripts (OTrs) in normal human tissues. Public raw RNA-seq data of normal tissues were downloaded from SRA (supplemental table S3) and were processed using the same pipeline as for lung and testis described in the material and methods section. Transcripts originating from the same TSS for a specific locus were summed, as described in the material and methods section. For WGBS, fastq files of normal cortex tissue were downloaded from SRA (supplemental table S3). Read quality control was performed using Trim-Galore v0.5.0 and FastQC v0.11.8. Technical replicates were summed. Read alignment and methylation calling were performed using Bismark v0.20.0. BAM file of liver (supplemental table S3) was downloaded from ENCODE. Methylation calling was performed using Bismark v0.20.0. For all other normal tissues, normalized hg38 data were downloaded from ENCODE (supplemental table S3).

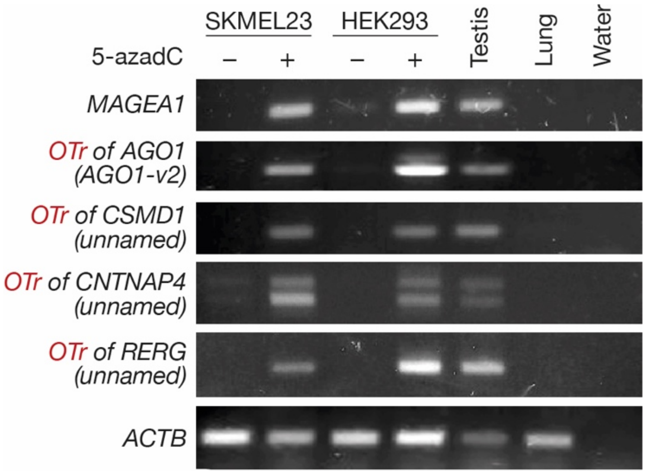

Fig. S7. Overlapping transcripts (OTr) qualified as "DNA methylation dependent" are induced following treatment with the DNA demethylating agent 5-azadC. Indicated cell lines were treated with 5-azadC (+) or vehicle (-), and were thereafter harvested for RT-PCR analyses. Expression analyses were performed for four OTrs, *MAGEA1* (5-azadC inducible control) and *ACTB* (internal standard). Testis (positive control) and lung (negative control) were included in the RT-PCR analysis.

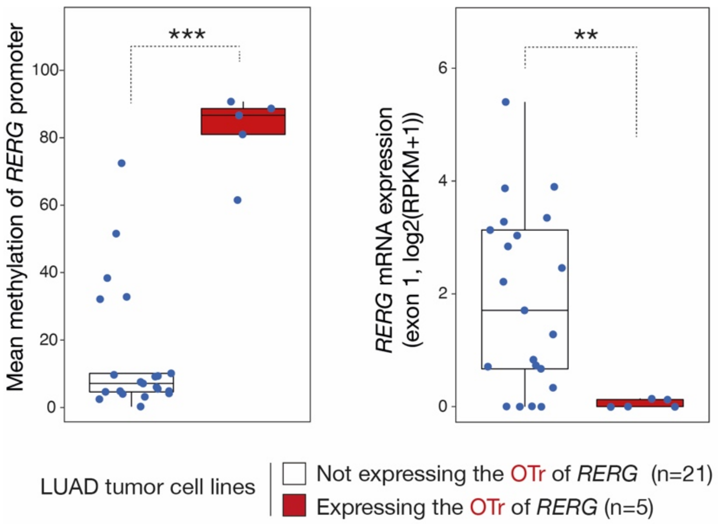

Fig. S8. Overlapping transcription is associated with *RERG* promoter hypermethylation and transcriptional downregulation. LUAD cell lines scoring positive for the expression of the overlapping transcript (OTr) of *RERG* (the OTr of *RERG* is later termed *CT-RERG*) were determined on the basis of RNA-seq data (TPM ≥ 1). Mean methylation level of RERG was determined by computing Methyl-seq methylation values for CpGs (n=55) located -/+ 400bp of TSS. Exon quantification (FeatureCounts v2.0.0) was used to determine the expression of *RERG* (specific exon 1). ** and *** Mann-Whitney test, *p*-value <0.01 and <0.001, respectively.

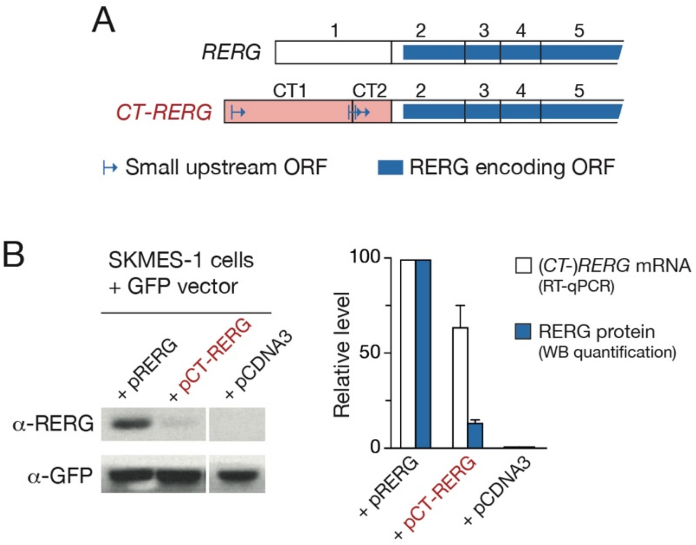

Fig. S9. Short upstream open reading frames (uORFs) in *CT-RERG* mRNA inhibit translation of the RERG protein. (*A*) Examination of the first two specific exons of *CT-RERG*, however, revealed the presence of three short upstream open reading frames (uORFs). Several reports have shown that uORFs can be associated with reduced protein expression by inhibiting translation initiation from the main downstream ORF (7,8). (*B*) We therefore compared the ability of *CT-RERG* and *RERG* transcripts to lead to production of the RERG protein. To this end, expression vectors carrying the corresponding cDNAs (pRERG and pCT-RERG), as well as the empty vector (pCDNA3), were transfected together with a GFP-expressing vector into the SKMES-1 lung tumor cell line. Western blotting (WB), performed at day 3 after transfection, examined the presence of the RERG protein and GFP as an internal control. Three experimental replicates were performed (one illustrative WB is shown). Band intensities in WBs were quantified with ImageJ. From the same transfected cell groups, we purified DNAse-treated RNA to check (*CT-*)*RERG* relative expression levels by RT-qPCR analysis (RT-minus subtraction and *ACTB* normalization were applied). The results revealed a marked decrease of RERG protein levels in p*CT-RERG* transfectants (14%), when compared with pRERG transfectants (taken as 100% reference). *CT-RERG* mRNA levels in p*CT-RERG* transfectants were only moderately lower (63%) than *RERG* mRNA level in pRERG transfectants. These data reveal therefore marked inhibition of RERG protein translation in *CT-RERG* transcripts. *Detailed protocol: RERG* and *CT-RERG* 5'-UTRs were amplified by PCR using the high fidelity PrimeStar HS DNA polymerase (Takara) with primers carrying a 5' overhang with a restriction site for *Hin*dIII (forward primer) and *Xho*I (reverse primer) (Table S2). PCR fragments were cloned into pcDNA3.1 expression vector (Invitrogen), and verified by sequencing. pTM624 GFP expressing vector was kindly provided by Thomas Michiels (de Duve Institute). SKMES-1 cells were plated at 150.000 cells/well of a 6w plate the day before transfection. Cells were transfected with 1.5 µg of plasmid pRERG, pCT-RERG or empty pcDNA3.1 and 0.5 µg of pTM624 using 6 µl Viafect transfection reagent (Promega) according to manufacturer's instructions. RNA (treated with Turbo DNAse, Invitrogen) and proteins were harvested 3 days post-transfection. For WB analysis, both membranes for RERG and GFP detection were blocked in 5% milk in TBS-Tween 0.1%. For detection of RERG protein, rabbit polyclonal anti-RERG antibody (10687-1-AP, Sanbio) was used at a final dilution of 1/5000 in 5% milk in TBS-Tween 0.1%. HRP-conjugated goat anti-rabbit secondary antibody (ADI-SAB-300-J, Enzo Life Sciences) antibody was used at 1/10000 in TBS-Tween 0.1%. For GFP protein detection, we used goat polyclonal anti-GFP antibody (ab6673, Abcam) at a final dilution of 1/5000 in 5% milk in TBS-Tween 0.1%. HRP-conjugated donkey anti-goat (PA1-28664, Life Technologies) was used at a final dilution 1/5000 in TBS-Tween 0.1%.

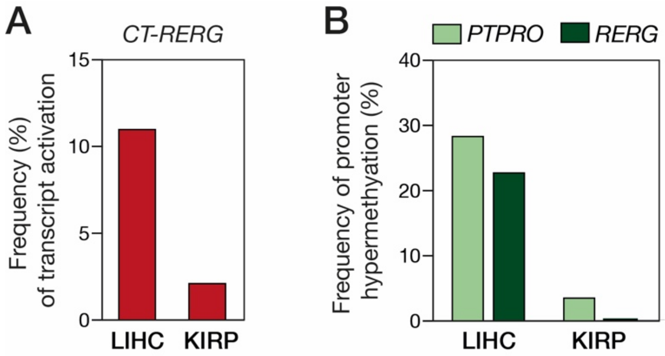

Fig. S10. Renal carcinoma shows lower frequency of both transcriptional activation of *CT-RERG* and hypermethylation of *PTPRO* and *RERG* downstream promoters. (*A*) The frequency of activation of *CT-RERG* transcription (TPM>1) was evaluated in liver hepatocarcinoma (LIHC, n=369) and renal papillary cell carcinoma (KIRP, n=273) through the analysis of RNA-seq data from the TCGA. (*B*) The frequency of hypermethylation of *PTPRO* and *RERG* promoters in these same tumor samples were evaluated through the analysis of Infinium methylation data from the TCGA (probe intensity ratio > 0.2, as explained in Fig.6H-I).

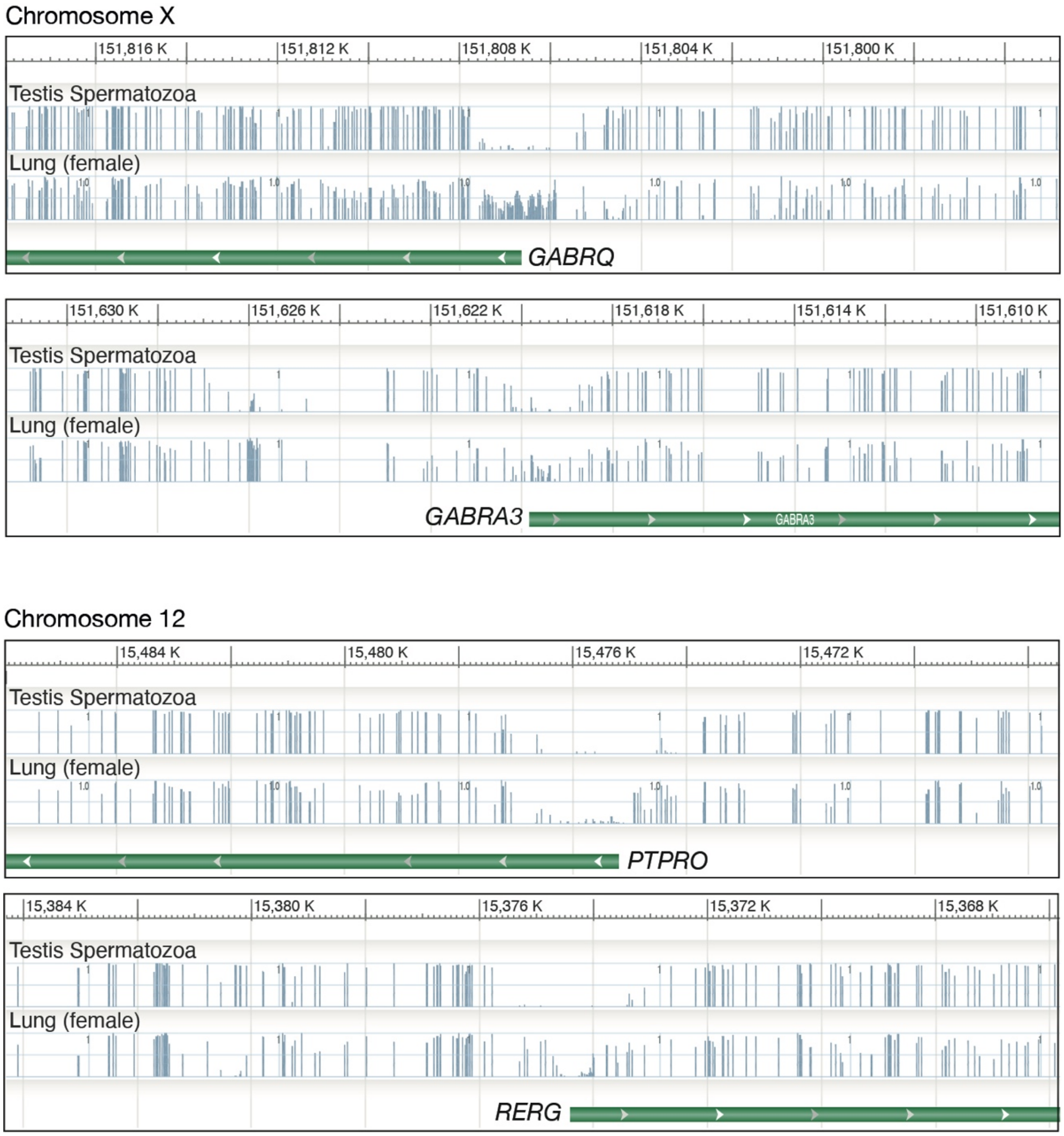

Fig. S11. Lack of hypermethylation of *GABRQ*/*GABRA3* and *PTPRO*/*RERG* promoters in testicular germ cells. Bisulfite-seq data from human testicular spermatozoa and normal lung were visualized with the NIH Roadmap epigenomics track viewer: histograms depict CpG methylation levels (fraction methylated). TSS position and orientation (green bar and arrowhead) of the four genes analyzed are indicated. Partial methylation of *GABRQ* and *GABRA3* gene promoters in the lung is attributable to the presence of an inactive X chromosome, as the tissue was obtained from a female individual.

Supplementary Tables

Table S1. Transcript references and TSS positions of overlapping transcripts (OTr) and overlapped promoters (OPr) identified in this study.

	Overlapping transcripts (OTr)					Overlapped promoter (Opr)			
Chromosome	NCBI Refseq description	Corresponding reference(s) in Ensembl (ENST) or in-house generated GTF file (MSTRG)	Position of TSS	Strand		Gene symbol (variant)	Corresponding ENST or MSTRG	Position of TSS	Strand
chrX	*CT-GABRA3*	MSTRG.42953.5, MSTRG.42953.6, MSTRG.42953.8, MSTRG.42953.4, MSTRG.42953.3, MSTRG.42953.15, MSTRG.42953.7, MSTRG.42953.13, MSTRG.42953.14, MSTRG.42953.12	152698719	-	->	*GABRQ*	ENST00000598523.2	152638185	+
						GABRA3	ENST00000370314.8	152451315	-
chr19	*Unnamed*	MSTRG.20900.2	38823300	+	->	*ECH1*	ENST00000221418.8	38832005	-
chr7	*Unnamed*	MSTRG.35963.2	5794482	+	->	*ZNF815P*	ENST00000421890.5	5823160	+
chr1	*AGO1-v2*	ENST00000373206.5	35869808	+	->	*AGO1*	ENST00000373204.5	35883209	+
chr12	*Unnamed (CT-RERG)*	MSTRG.8305.2	15348675	-	->	*RERG*	ENST00000256953.6	15221417	-
chr14	*LINC00221*	ENST00000603633.2	106482439	+	->	*IGHVII-44-2*	ENST00000517728.1	106494383	-
chr16	*Unnamed*	MSTRG.16001.1	76235202	+	->	*CNTNAP4*	ENST00000611870.4	76277278	+
chr8	*Unnamed*	MSTRG.37947.3	3183689	-	->	*CSMD1 (vX)*	ENST00000520561.1	3113363	-
chr6	*Unnamed*	MSTRG.35314.8	122482079	+	->	*PKIB*	ENST00000368452.6	122610232	+
chr19	*Unnamed*	MSTRG.20651.2	32700158	-	->	*AC008736.1*	ENST00000592431.1	32691750	-
chr19	*Unnamed*	MSTRG.20651.2	32700158	-	->	*NUDT19*	ENST00000397061.3	32691961	+
chr11	*Unnamed*	MSTRG.7040.15	73607387	-	->	*FAM168A*	ENST00000064778.8	73598189	-
chr12	*Unnamed*	MSTRG.8692.1	48854530	+	->	*FKBP11*	ENST00000550765.5	48925827	-
chr15	*Unnamed*	ENST00000558873.1	65300618	-	->	*RNU5A-1*	ENST00000362698.1	65296051	+
chr15	*Unnamed*	MSTRG.13615.1	66582190	+	->	*SMAD6*	ENST00000557916.5	66703259	+
chr16	*Unnamed*	MSTRG.15775.2	68527848	+	->	*ZFP90 V1*	ENST00000563169.6	68539284	+
chr6	*Unnamed*	MSTRG.34416.1	33246857	+	->	*HCG25*	ENST00000450514.5	33249534	+
chr1	*Unnamed*	MSTRG.191.7	6427871	+	->	*ESPN (vX)*	ENST00000418286.1	6440378	+
chr7	*DDC*	ENST00000444124.6, MSTRG.36458.11	50565457	-	->	*DDC (vX)*	MSTRG.36458.12	50468168	-
chr20	*GNAS-v4*	ENST00000371075.7	58839718	+	->	*GNAS*	ENST00000603546.1	58892333	+
chr1	*AL590666.3*	ENST00000448869.1	156661424	-	->	*BCAN*	ENST00000329117.9	156641948	+
chrX	*ZC4H2-v2*	ENST00000337990.2	65034713	-	->	*ZC4H2*	ENST00000374839.7	64976484	-
chr1	*Unnamed*	MSTRG.3292.8	204377853	-	->	*PLEKHA6*	ENST00000272203.7	204359916	-
chr20	*EPB41L1-v5*	ENST00000202028.9	36091504	+	->	*EPB41L1*	ENST00000338074.7	36154740	+
chr3	*Unnamed*	MSTRG.28969.12	129003864	-	->	*EFCC1*	ENST00000436022.2	129001629	+
chr15	*ISL2*	ENST00000290759.8	76336724	+	->	*AC027243.1*	ENST00000559539.1	76342063	-
chr17	*Unnamed*	MSTRG.17424.1	38419280	+	->	*ARHGAP23*	ENST00000622683.4	38428418	+
chr17	*IGF2BP1-v1*	ENST00000290341.7	48997171	+	->	*IGF2BP1 (vX)*	ENST00000499130.6	49013917	+
chr11	*WT1-vA*	ENST00000332351.7	32435630	-	->	*WT1 (vE)*	ENST00000527775.1	32429029	-
						WT1 (vG)	ENST00000526685.1	32400615	-
chr16	*FBXL16*	ENST00000397621.5	705829	-	->	*FBXL16 (vX)*	ENST00000562585.1	695263	-
chr11	*PAX6-v1*	ENST00000640335.1, ENST00000638278.1, ENST00000379107.7, ENST00000379109.7, ENST00000640617.1, ENST00000640684.1, ENST00000638250.1, ENST00000639394.1, ENST00000640242.1, ENST00000639054.1, ENST00000527769.5, ENST00000640613.1, ENST00000471303.6, ENST00000606377.6	31813131	-	->	*PAX6 (vX)*	ENST00000640735.1	31801992	-
chr15	*CPEB1-v1*	ENST00000615198.4	82647977	-	->	*CPEB1 (v2)*	ENST00000562833.2	82571447	-
chr2	*WNT10A*	ENST00000258411.7	218880363	+	->	*WNT10A (vX)*	ENST00000489887.1	218893221	+
chr2	*GAD1-v67*	ENST00000358196.8	170816114	+	->	*GAD1 (vX)*	ENST00000486850.1	170822087	+
chr17	*Unnamed*	MSTRG.17685.2, ENST00000588097.5, MSTRG.17685.5	44315335	-	->	*RUNDC3A*	ENST00000590834.5	44308413	+
						RUNDC3A-AS1	ENST00000586388.1	44308393	-
chr19	*AC005775.1*	ENST00000592413.2	507833	-	->	*MADCAM1 (vX)*	ENST00000622449.2	497817	+
chr5	*NRG2-v1*	ENST00000361474.5	140043299	-	->	*NRG2 (vX1)*	ENST00000340391.7	139904397	-
						AC008667.2	ENST00000504413.1	139848290	+

A merged GTF file was generated following *de novo* assembling with StringTie of RNA-seq alignments derived from LUAD cell lines and normal human tissues. Besides transcripts that had been previously described in Ensembl (ENST), novel undescribed transcripts (MSTRG) were identified. For some OTrs, multiple splice variants, all starting from the same promoter, were detected.

Table S2. List of primers and PCR conditions.

	Forward primer (5'-3')	Reverse primer (5'-3')	Additional information
**PCR**			
ACTINE-β (ACTB)	CCCTGGACTTCGAGCAAGAGAT	AAGGTAGTTTCGTGGATGCCACA	DreamTaq (20 cycles / Annealing : 60°C and 30 sec / Elongation : 72°C and 30 sec)
GABRA3	GGGCTCAACCTCCAACTT	CATCATGCCATGTCTGCCGAAA	DreamTaq (35 cycles / Annealing : 60°C and 30 sec / Elongation : 72°C and 30 sec)
CT-GABRA3	CTGGGAGACAGAGCGAGA	CATCATGCCATGTCTGCCGAAA	DreamTaq (37 cycles / Annealing : 60°C and 30 sec / Elongation : 72°C and 1 min)
MAGEA1	GCCGAAGGAACCTGACC	ACTGGGTTGCCTCTGTCG	DreamTaq (34 cycles / Annealing : 60°C and 30 sec / Elongation : 72°C and 30 sec)
RERG	CTCGCAAACGCAACCTGAA	GAAATCTCACTACAAGAGCTGACTT	DreamTaq (35 cycles / Annealing : 58°C and 30 sec / Elongation : 72°C and 30 sec)
CT-RERG	TCCTTCACTGTTGTTGGCAGAA	GAAATCTCACTACAAGAGCTGACTT	DreamTaq (37 cycles / Annealing : 58°C and 30 sec / Elongation : 72°C and 30 sec)
AGO1	GCAAGCGCCTCAGGAGTGCGT	CTTGCGATCACCAAAGATCTGA	DreamTaq (34 cycles / Annealing : 60°C and 30 sec / Elongation : 72°C and 30 sec)
CSMD1	GGATCACGAGGCACCACAGTA	CCATGTACCGATCTCCGATT	DreamTaq (33 cycles / Annealing : 60°C and 30 sec / Elongation : 72°C and 30 sec)
CNTNAP4	ACAACCTCTTCAGCACAACCT	CCACTATCACTGAACATCA	DreamTaq (35 cycles / Annealing : 60°C and 30 sec / Elongation : 72°C and 30 sec)
**qPCR**			
(CT-)RERG	GAAGTGCTGCCACTTAAGAACA	GACGCACCTCTCGACACAAT	KAPA SYBR FAST (Annealing /Elongation : 60°C and 30 sec)
**Bisulfite-converted DNA**			
CT-GABRA3 first PCR	TYGATTTTTTATTTAGGTAGAATTT	TAAAATAATAACRACCCAACCTAA	DreamTaq (35 cycles / Annealing : 55°C and 30 sec / Elongation : 72°C and 30 sec)
CT-GABRA3 second PCR	ATTTAGGTAGAATTTAGTTTTTAT	CCCTACRAAATAACCCAAA	DreamTaq (35 cycles / Annealing : 55°C and 30 sec / Elongation : 72°C and 30 sec)
GABRA3 first PCR	AGTGGTGAATTTAAAGTTAGTAAAGG	CCCCAATATCTCCCTACTCAAAT	DreamTaq (35 cycles / Annealing : 55°C and 30 sec / Elongation : 72°C and 30 sec)
GABRA3 second PCR	GATAGAGAGGGAGGGAGGTAGA	CTAAACTTCCACCAACCCCACT	DreamTaq (35 cycles / Annealing : 55°C and 30 sec / Elongation : 72°C and 30 sec)
**MS-qPCR**			
GABRA3-MSM	AGCGAGAGAGCGTGAGCGC	TCTCTCCTAATCCCTCTCTCG	Takyon™ Rox SYBR Core Kit (Annealing : 60°C and 30 sec / Elongation 72°C and 30 sec)
GABRA3-MSU	AGTGAGAGAGTGTGAGTGT	TCTCTCCTAATCCCTCTCTCA	Takyon™ Rox SYBR Core Kit (Annealing : 60°C and 30 sec / Elongation 72°C and 30 sec)
CT-GABRA3-MSM	GTTGAGGGAGGATTGAGGC	CTACGAAATAACCCAAACCCG	Takyon™ Rox SYBR Core Kit (Annealing : 60°C and 30 sec / Elongation 72°C and 45 sec)
CT-GABRA3-MSU	GTTGAGGGAGGATTGAGGT	CTACAAAATAACCCAAACCCA	Takyon™ Rox SYBR Core Kit (Annealing : 60°C and 30 sec / Elongation 72°C and 45 sec)
**pcDNA3 Cloning**			
RERG (HindIII & Xho1)	ATGAAAGCTTGCAACTCGCAAACGCAAC	ATGACTCGAGCTAACTACTGATTTTGGTGAGC	PrimeSTAR (37 cycles / Annealing : 62°C and 10 sec / Elongation : 72°C and 1 min)
CT-RERG (HindIII & Xho1)	ATGAAAGCTTGTTTCACTGTGTTAGCCAG	ATGACTCGAGCTAACTACTGATTTTGGTGAGC	PrimeSTAR (37 cycles / Annealing : 62°C and 10 sec / Elongation : 72°C and 1 min)

Table S3. Transcriptomic and Methylomic datasets used in this study.

BioSample	Name	Accession number	BioProject	Layout	Instrument	File	Citation
			**RNA-seq datasets**				
LUAD cell line	A427	DRR016694	PRJDB2256	PAIRED	Illumina HiSeq 2500	Fastq	DBTSS
LUAD cell line	A549	DRR016695	PRJDB2256	PAIRED	Illumina HiSeq 2500	Fastq	DBTSS
LUAD cell line	ABC1	DRR016696	PRJDB2256	PAIRED	Illumina HiSeq 2500	Fastq	DBTSS
LUAD cell line	H1299	DRR016697	PRJDB2256	PAIRED	Illumina HiSeq 2500	Fastq	DBTSS
LUAD cell line	H1437	DRR016698	PRJDB2256	PAIRED	Illumina HiSeq 2500	Fastq	DBTSS
LUAD cell line	H1648	DRR016699	PRJDB2256	PAIRED	Illumina HiSeq 2500	Fastq	DBTSS
LUAD cell line	H1650	DRR016700	PRJDB2256	PAIRED	Illumina HiSeq 2500	Fastq	DBTSS
LUAD cell line	H1703	DRR016701	PRJDB2256	PAIRED	Illumina HiSeq 2500	Fastq	DBTSS
LUAD cell line	H1819	DRR016702	PRJDB2256	PAIRED	Illumina HiSeq 2500	Fastq	DBTSS
LUAD cell line	H1975	DRR016703	PRJDB2256	PAIRED	Illumina HiSeq 2500	Fastq	DBTSS
LUAD cell line	H2126	DRR016704	PRJDB2256	PAIRED	Illumina HiSeq 2500	Fastq	DBTSS
LUAD cell line	H2228	DRR016705	PRJDB2256	PAIRED	Illumina HiSeq 2500	Fastq	DBTSS
LUAD cell line	H2347	DRR016706	PRJDB2256	PAIRED	Illumina HiSeq 2500	Fastq	DBTSS
LUAD cell line	H322	DRR016707	PRJDB2256	PAIRED	Illumina HiSeq 2500	Fastq	DBTSS
LUAD cell line	II18	DRR016708	PRJDB2256	PAIRED	Illumina HiSeq 2500	Fastq	DBTSS
LUAD cell line	LC2ad	DRR016709	PRJDB2256	PAIRED	Illumina HiSeq 2500	Fastq	DBTSS
LUAD cell line	PC14	DRR016710	PRJDB2256	PAIRED	Illumina HiSeq 2500	Fastq	DBTSS
LUAD cell line	PC3	DRR016711	PRJDB2256	PAIRED	Illumina HiSeq 2500	Fastq	DBTSS
LUAD cell line	PC7	DRR016712	PRJDB2256	PAIRED	Illumina HiSeq 2500	Fastq	DBTSS
LUAD cell line	PC9	DRR016713	PRJDB2256	PAIRED	Illumina HiSeq 2500	Fastq	DBTSS
LUAD cell line	RERFLC-Ad1	DRR016714	PRJDB2256	PAIRED	Illumina HiSeq 2500	Fastq	DBTSS
LUAD cell line	RERFLC-Ad2	DRR016715	PRJDB2256	PAIRED	Illumina HiSeq 2500	Fastq	DBTSS
LUAD cell line	RERFLC-KJ	DRR016716	PRJDB2256	PAIRED	Illumina HiSeq 2500	Fastq	DBTSS
LUAD cell line	RERFLC-MS	DRR016717	PRJDB2256	PAIRED	Illumina HiSeq 2500	Fastq	DBTSS
LUAD cell line	RERFLC-OK	DRR016718	PRJDB2256	PAIRED	Illumina HiSeq 2500	Fastq	DBTSS
LUAD cell line	VMRC-LCD	DRR016719	PRJDB2256	PAIRED	Illumina HiSeq 2500	Fastq	DBTSS
Normal tissue	Lung	SRR577579 SRR577582	PRJNA34535	PAIRED	Illumina HiSeq 2000	Fastq	NIH Roadmap epigenomics
Normal tissue	Testis	ERR315352 ERR315415 ERR315492	PRJEB4337	PAIRED	Illumina HiSeq 2000	Fastq	Science for Life Laboratory, Stockholm, Sweden
Normal tissue	Esophagus	ERR315411 ERR315398 ERR315489 ERR315434 ERR315472 ERR315362	PRJEB4337	PAIRED	Illumina HiSeq 2000	Fastq	Science for Life Laboratory, Stockholm, Sweden
Normal tissue	Colon	ERR315357 ERR315484 ERR315462 ERR315400 ERR315348 ERR315403	PRJEB4337	PAIRED	Illumina HiSeq 2000	Fastq	Science for Life Laboratory, Stockholm, Sweden
Normal tissue	Adipose_tissue	ERR315332 ERR315431 ERR315343 ERR315342 ERR315378	PRJEB4337	PAIRED	Illumina HiSeq 2000	Fastq	Science for Life Laboratory, Stockholm, Sweden
Normal tissue	Cerebral_cortex	ERR315455 ERR315432 ERR315477	PRJEB4337	PAIRED	Illumina HiSeq 2000	Fastq	Science for Life Laboratory, Stockholm, Sweden
Normal tissue	Heart	ERR315384 ERR315328 ERR315356 ERR315367 ERR315413 ERR315435 ERR315389 ERR315331 ERR315430	PRJEB4337	PAIRED	Illumina HiSeq 2000	Fastq	Science for Life Laboratory, Stockholm, Sweden
Normal tissue	Liver	ERR315327 ERR315414 ERR315463 ERR315394 ERR315451	PRJEB4337	PAIRED	Illumina HiSeq 2000	Fastq	Science for Life Laboratory, Stockholm, Sweden
Normal tissue	Pancreas	ERR315466 ERR315436 ERR315479 ERR315429	PRJEB4337	PAIRED	Illumina HiSeq 2000	Fastq	Science for Life Laboratory, Stockholm, Sweden
Normal tissue	Placenta	ERR315374 ERR315478 ERR315336 ERR315375 ERR315476 ERR315377 ERR315399	PRJEB4337	PAIRED	Illumina HiSeq 2000	Fastq	Science for Life Laboratory, Stockholm, Sweden
Normal tissue	Skin	ERR315339 ERR315401 ERR315460 ERR315376 ERR315372 ERR315464	PRJEB4337	PAIRED	Illumina HiSeq 2000	Fastq	Science for Life Laboratory, Stockholm, Sweden
Normal tissue	Stomach	ERR315379 ERR315369 ERR315467 ERR315485	PRJEB4337	PAIRED	Illumina HiSeq 2000	Fastq	Science for Life Laboratory, Stockholm, Sweden
			**DNA Methylation datasets**				
LUAD cell line	A427	bs_data_9606_A427	DBTSS	PAIRED	Illumina HiSeq 2500	BED	DBTSS
LUAD cell line	A549	bs_data_9606_A549	DBTSS	PAIRED	Illumina HiSeq 2500	BED	DBTSS
LUAD cell line	ABC1	bs_data_9606_ABC1	DBTSS	PAIRED	Illumina HiSeq 2500	BED	DBTSS
LUAD cell line	H1299	bs_data_9606_H1299	DBTSS	PAIRED	Illumina HiSeq 2500	BED	DBTSS
LUAD cell line	H1437	bs_data_9606_H1437	DBTSS	PAIRED	Illumina HiSeq 2500	BED	DBTSS
LUAD cell line	H1648	bs_data_9606_H1648	DBTSS	PAIRED	Illumina HiSeq 2500	BED	DBTSS
LUAD cell line	H1650	bs_data_9606_H1650	DBTSS	PAIRED	Illumina HiSeq 2500	BED	DBTSS
LUAD cell line	H1703	bs_data_9606_H1703	DBTSS	PAIRED	Illumina HiSeq 2500	BED	DBTSS
LUAD cell line	H1819	bs_data_9606_H1819	DBTSS	PAIRED	Illumina HiSeq 2500	BED	DBTSS
LUAD cell line	H1975	bs_data_9606_H1975	DBTSS	PAIRED	Illumina HiSeq 2500	BED	DBTSS
LUAD cell line	H2126	bs_data_9606_H2126	DBTSS	PAIRED	Illumina HiSeq 2500	BED	DBTSS
LUAD cell line	H2228	bs_data_9606_H2228	DBTSS	PAIRED	Illumina HiSeq 2500	BED	DBTSS
LUAD cell line	H2347	bs_data_9606_H2347	DBTSS	PAIRED	Illumina HiSeq 2500	BED	DBTSS
LUAD cell line	H322	bs_data_9606_H322	DBTSS	PAIRED	Illumina HiSeq 2500	BED	DBTSS
LUAD cell line	II18	bs_data_9606_II18	DBTSS	PAIRED	Illumina HiSeq 2500	BED	DBTSS
LUAD cell line	LC2ad	bs_data_9606_LC2ad	DBTSS	PAIRED	Illumina HiSeq 2500	BED	DBTSS
LUAD cell line	PC14	bs_data_9606_PC14	DBTSS	PAIRED	Illumina HiSeq 2500	BED	DBTSS
LUAD cell line	PC3	bs_data_9606_PC3	DBTSS	PAIRED	Illumina HiSeq 2500	BED	DBTSS
LUAD cell line	PC7	bs_data_9606_PC7	DBTSS	PAIRED	Illumina HiSeq 2500	BED	DBTSS
LUAD cell line	PC9	bs_data_9606_PC9	DBTSS	PAIRED	Illumina HiSeq 2500	BED	DBTSS
LUAD cell line	RERFLC-Ad1	bs_data_9606_RERFLC-Ad1	DBTSS	PAIRED	Illumina HiSeq 2500	BED	DBTSS
LUAD cell line	RERFLC-Ad2	bs_data_9606_RERFLC-Ad2	DBTSS	PAIRED	Illumina HiSeq 2500	BED	DBTSS
LUAD cell line	RERFLC-KJ	bs_data_9606_RERFLC-KJ	DBTSS	PAIRED	Illumina HiSeq 2500	BED	DBTSS
LUAD cell line	RERFLC-MS	bs_data_9606_RERFLC-KJ	DBTSS	PAIRED	Illumina HiSeq 2500	BED	DBTSS
LUAD cell line	RERFLC-OK	bs_data_9606_RERFLC-OK	DBTSS	PAIRED	Illumina HiSeq 2500	BED	DBTSS
LUAD cell line	VMRC-LCD	bs_data_9606_VMRC-LCD	DBTSS	PAIRED	Illumina HiSeq 2500	BED	DBTSS
Normal tissue	Lung	GSM983647	NIH Roadmap epigenomics	PAIRED	Illumina HiSeq 2000	BED	NIH Roadmap epigenomics
Normal tissue	Sperm	GSM1127119	NIH Roadmap epigenomics	PAIRED	Illumina HiSeq 2000	BED	NIH Roadmap epigenomics
Normal tissue	Esophagus	ENCFF625GVK	NIH Roadmap epigenomics	PAIRED	Illumina HiSeq 2000	BED	NIH Roadmap epigenomics
Normal tissue	Colon	ENCFF157POM	NIH Roadmap epigenomics	PAIRED	Illumina HiSeq 2000	BED	NIH Roadmap epigenomics
Normal tissue	Adipose_tissue	ENCFF318AMC	NIH Roadmap epigenomics	PAIRED	Illumina HiSeq 2000	BED	NIH Roadmap epigenomics
Normal tissue	Heart	ENCFF536RSX	NIH Roadmap epigenomics	PAIRED	Illumina HiSeq 2000	BED	NIH Roadmap epigenomics
Normal tissue	Pancreas	ENCFF763RUE	NIH Roadmap epigenomics	PAIRED	Illumina HiSeq 2000	BED	NIH Roadmap epigenomics
Normal tissue	Placenta	ENCFF4370KM	NIH Roadmap epigenomics	PAIRED	Illumina HiSeq 2000	BED	NIH Roadmap epigenomics
Normal tissue	Skin	ENCFF219GCQ	NIH Roadmap epigenomics	PAIRED	Illumina HiSeq 2000	BED	NIH Roadmap epigenomics
Normal tissue	Stomach	ENCFF49700	NIH Roadmap epigenomics	PAIRED	Illumina HiSeq 2000	BED	NIH Roadmap epigenomics
Normal tissue	Liver	ENCFF356KGQ	NIH Roadmap epigenomics	SINGLE	Illumina HiSeq 2500	BAM	NIH Roadmap epigenomics
Normal tissue	Cerebral_cortex	SRR3278486 , SRR3278482	Johns Hopkins Center for Epigenetics	PAIRED	Illumina HiSeq 2000	Fastq	Johns Hopkins Center for Epigenetics

Original uncropped images

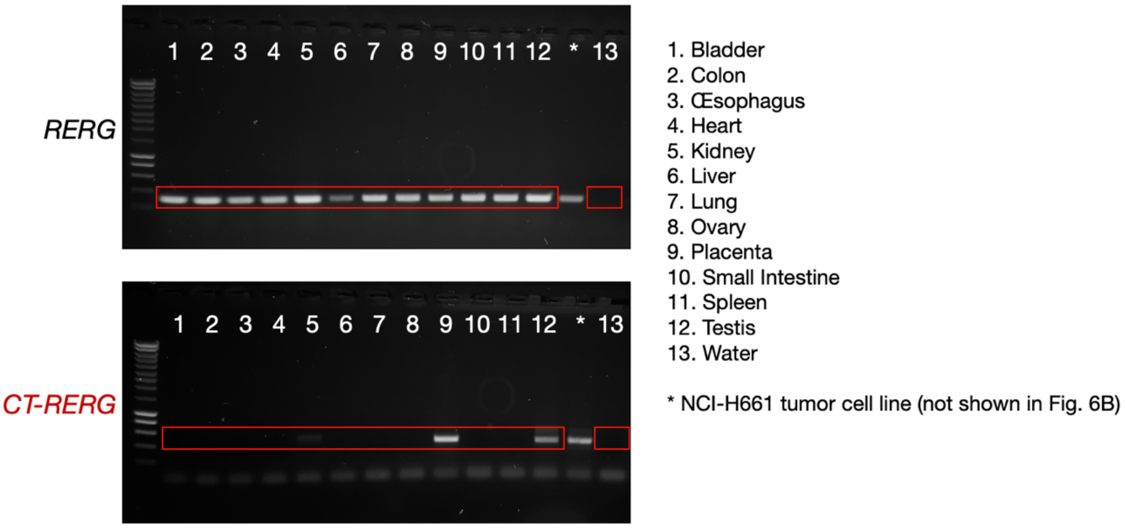

Original images of Fig. 6B. Image parts included in the manuscript figure are delimited by red boxes.

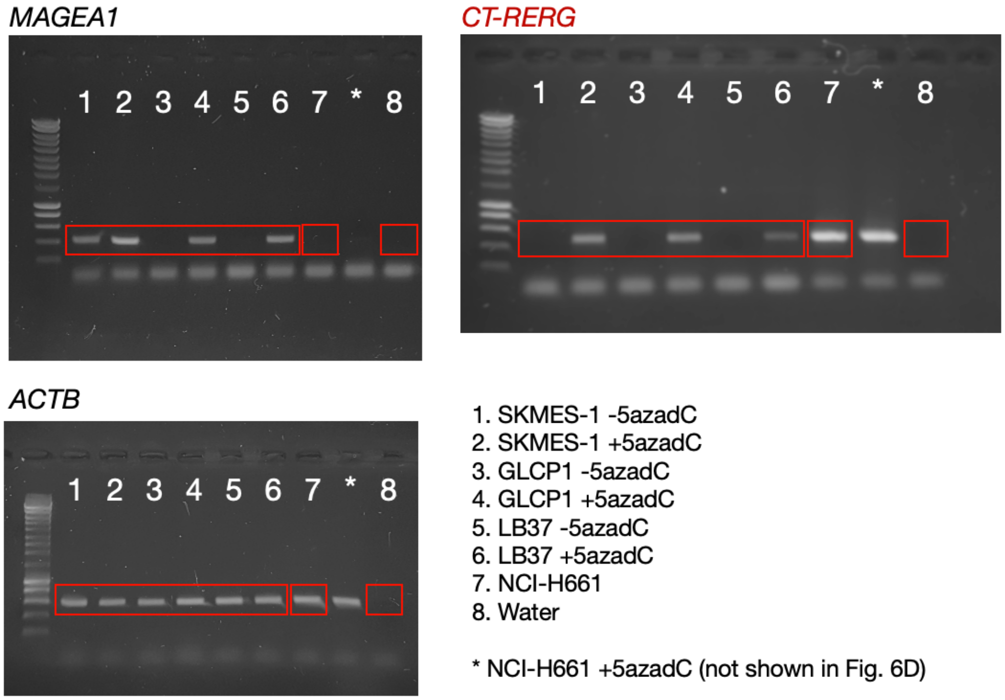

Original images of Fig. 6D. Image parts included in the manuscript figure are delimited by red boxes.

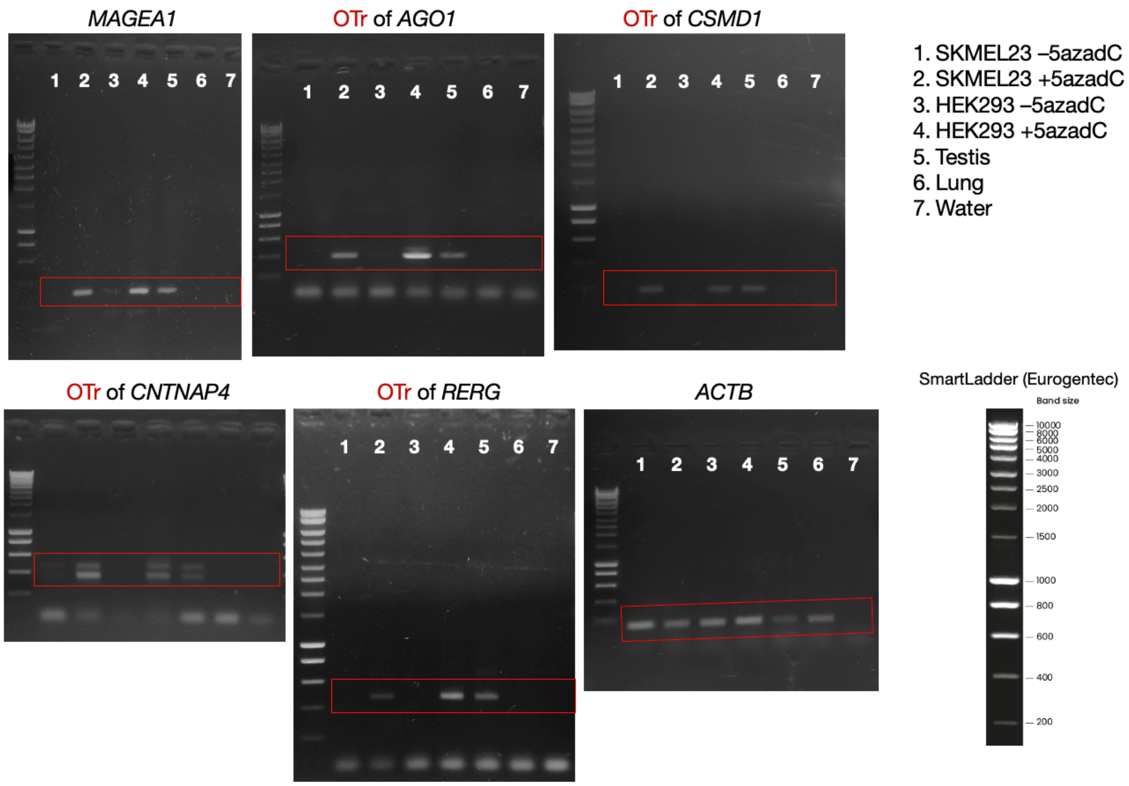

Original images of Fig. S7. Image parts included in the supplementary figure are delimited by red boxes.

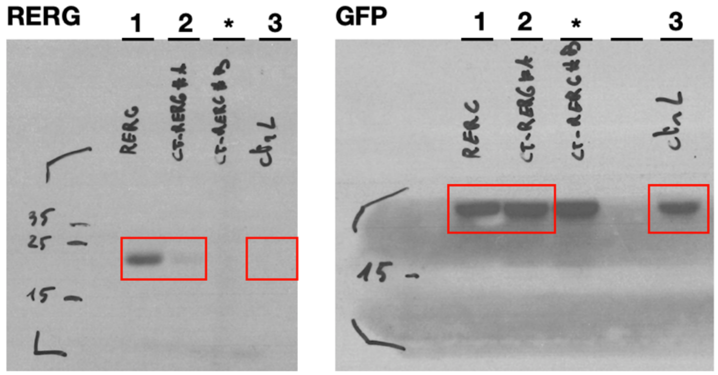

1. +pRERG
2. +pCT-RERG
3. +pCDNA3

* +pCT-RERG#B (not shown in Fig S6B)

Original images of Fig. S9B. Image parts included in the supplementary figure are delimited by red boxes. Considering the similar size of RERG and GFP proteins, two separate Western blots with identical amounts of indicated protein samples were performed. pCT-RERG#B contains a cDNA that corresponds to an alternative and less abundant splicing variant of *CT-RERG* (lacking exon 2 of *RERG*).

Supplementary References

1. Andrews, S.D. (2010) FastQC: a quality control tool for high throughput sequence data., **Available online at: http://www.bioinformatics.babraham.ac.uk/projects/fastqc**.
2. Bolger, A.M., Lohse, M. and Usadel, B. (2014) Trimmomatic: a flexible trimmer for Illumina sequence data. *Bioinformatics*, **30**, 2114-2120.
3. Kim, D., Langmead, B. and Salzberg, S.L. (2015) HISAT: a fast spliced aligner with low memory requirements. *Nat Methods*, **12**, 357-360.
4. Li, H., Handsaker, B., Wysoker, A., Fennell, T., Ruan, J., Homer, N., Marth, G., Abecasis, G., Durbin, R. and Genome Project Data Processing, S. (2009) The Sequence Alignment/Map format and SAMtools. *Bioinformatics*, **25**, 2078-2079.
5. Robinson, J.T., Thorvaldsdottir, H., Winckler, W., Guttman, M., Lander, E.S., Getz, G. and Mesirov, J.P. (2011) Integrative genomics viewer. *Nat Biotechnol*, **29**, 24-26.
6. Pertea, M., Pertea, G.M., Antonescu, C.M., Chang, T.C., Mendell, J.T. and Salzberg, S.L. (2015) StringTie enables improved reconstruction of a transcriptome from RNA-seq reads. *Nat Biotechnol*, **33**, 290-295.
7. Calvo, S.E., Pagliarini, D.J. and Mootha, V.K. (2009) Upstream open reading frames cause widespread reduction of protein expression and are polymorphic among humans. *Proc Natl Acad Sci U S A*, **106**, 7507-7512.
8. Morris, D.R. and Geballe, A.P. (2000) Upstream open reading frames as regulators of mRNA translation. *Mol Cell Biol*, **20**, 8635-8642.
